# FOSL1 Inhibits Type I Interferon Responses to Malaria and Viral Infections by Blocking TBK1 and TRAF3/TRIF Interactions

**DOI:** 10.1128/mBio.02161-16

**Published:** 2017-01-03

**Authors:** Baowei Cai, Jian Wu, Xiao Yu, Xin-zhuan Su, Rong-Fu Wang

**Affiliations:** a State Key Laboratory of Cellular Stress Biology, Innovation Center for Cell Biology, School of Life Sciences, Xiamen University, Xiamen, Fujian, People’s Republic of China; b Center for Inflammation and Epigenetics, Houston Methodist Research Institute, Houston, Texas, USA; c Laboratory of Malaria and Vector Research, National Institute of Allergy and Infectious Diseases, National Institutes of Health, Bethesda, Maryland, USA; d Department of Microbiology and Immunology, Weill Cornell Medicine, Cornell University, New York, New York, USA; e Institute of Biosciences and Technology, College of Medicine, Texas A & M University, Houston, Texas, USA.; Washington University School of Medicine

## Abstract

Innate immune response plays a critical role in controlling invading pathogens, but such an immune response must be tightly regulated. Insufficient or overactivated immune responses may lead to harmful or even fatal consequences. To dissect the complex host-parasite interactions and the molecular mechanisms underlying innate immune responses to infections, here we investigate the role of FOS-like antigen 1 (FOSL1) in regulating the host type I interferon (IFN-I) response to malaria parasite and viral infections. FOSL1 is known as a component of a transcription factor but was recently implicated in regulating the IFN-I response to malaria parasite infection. Here we show that FOSL1 can act as a negative regulator of IFN-I signaling. Upon stimulation with poly(I:C), malaria parasite-infected red blood cells (iRBCs), or vesicular stomatitis virus (VSV), FOSL1 “translocated” from the nucleus to the cytoplasm, where it inhibited the interactions between TNF receptor-associated factor 3 (TRAF3), TIR domain-containing adapter inducing IFN-β (TRIF), and Tank-binding kinase 1 (TBK1) via impairing K63-linked polyubiquitination of TRAF3 and TRIF. Importantly, FOSL1 knockout chimeric mice had lower levels of malaria parasitemia or VSV titers in peripheral blood and decreased mortality compared with wild-type (WT) mice. Thus, our findings have identified a new role for FOSL1 in negatively regulating the host IFN-I response to malaria and viral infections and have identified a potential drug target for controlling malaria and other diseases.

## IMPORTANCE

Infections of pathogens can trigger vigorous host immune responses, including activation and production of type I interferon (IFN-I). In this study, we investigated the role of FOSL1, a molecule previously known as a transcription factor, in negatively regulating IFN-I responses to malaria and viral infections. We showed that FOSL1 was upregulated and translocated into the cytoplasm of cells after stimulation for IFN-I production. FOSL1 could affect TRAF3 and TRIF ubiquitination and consequently impaired the association of TRAF3, TRIF, and TBK1, leading to inhibition of IFN-I signaling. *In vivo* experiments with FOSL1 knockout chimeric mice further validated the negative role of FOSL1 in IFN-I production and antimicrobial responses. This report reveals a new functional role for FOSL1 in IFN-I signaling and dissects the mechanism by which FOSL1 regulates IFN-I responses to malaria and viral infections, which can be explored as a potential drug target for disease control and management.

## INTRODUCTION

Innate immunity serves as the first line of host defense against invading pathogens and relies on the recognition of pathogen-associated molecular patterns (PAMPs) such as lipopolysaccharide (LPS), DNA, RNA, and carbohydrates from invading pathogens by pattern recognition receptors (PRRs) to activate the innate immune response (1, 2). In recent years, many PRRs have been identified, including retinoic acid-inducible gene I (RIG-I), melanoma differentiation-associated gene 5 (MDA5), cyclic GMP-AMP synthase (cGAS), Toll-like receptors (TLRs), and NOD-like receptors (NLRs) (1, 3–8). Activation of these PRRs recruits various adaptors, such as stimulator of interferon genes (STING, also known as MPYS, MITA, and Eris), mitochondrial antiviral signaling protein (MAVS, also called as Cardif, VISA, and IPS-I), and TIR domain-containing adapter inducing beta interferon (IFN-β) (TRIF), to directly interact with TNF receptor-associated factor 3 (TRAF3) and trigger auto-ubiquitination of TRAF3 (9–12). Ubiquitinated TRAF3 then interacts with Tank-binding kinase 1 (TBK1) to activate the transcription factor interferon-regulatory factor 3 (IRF3)-mediated type I interferon (IFN-I) signaling and antipathogen immune responses (13). However, an uncontrolled innate immune response can lead to redundant production of IFN-I and proinflammatory cytokines and cause autoimmune diseases, such as systemic lupus erythematosus (SLE) (14). Thus, production of IFN-I and other cytokines after pathogen infection needs to be appropriately regulated in order to eliminate invading pathogens while avoiding immune disorders (3, 15).

FOSL1 belongs to a gene family that consists of four members, namely, *FOS*, *FOSB*, *FOSL1*, and *FOSL2*, all encoding proteins containing leucine zippers (16). The members of the FOS transcription factor family are known to differentially regulate trophoblast migration and invasion (17). FOSL1 is also a member of the activator protein 1 (AP-1) complex containing Jun (c-jun, junB, junD), Fos (c-fos, fosB, fra-1, fra-2), activating transcription factor (ATF), and musculoaponeurotic fibrosarcoma (MAF) (16) and contributes to different cellular processes such as proliferation, differentiation, and apoptosis. FOSL1 acts as a key downstream effector of the phosphatidylinositol 3-kinase (PI3K)/AKT signaling pathway and is responsible for the development of trophoblast lineages (18), in addition to its role in regulating *Mmp9* gene expression (18). Recent studies showed that histone deacetylases 1, 2, and 3 are recruited to the regulatory and coding regions of the induced *Fosl1 (fra-1)* gene (19). Additionally, FOSL1 has been reported to play a role in various cancers (20). However, these studies mostly focused on the transcription factor activity of FOSL1 in the nucleus; its function in the cytoplasm, especially in regulating the IFN-I response during the host innate immune response to pathogen infection, remains unknown.

In this report, we show that, after stimulation with poly(I:C) or malaria parasite-infected red blood cells (iRBCs), FOSL1 was “translocated” from the nucleus to the cytoplasm, where it interacted with TRAF3 and TRIF to reduce IRF3 phosphorylation and IFN-I signaling. We further show that FOSL1 negatively regulated IFN-I response by reducing K63 ubiquitination of TRAF3/TRIF and blocking interaction of TRAF3/TRIF with TBK1. Our findings identify a previously unrecognized role of FOSL1 in negatively regulating IFN-I signaling. These molecular interactions can be exploited as potential targets for the treatment of pathogen infections and, perhaps, autoimmune diseases.

## RESULTS

### Enhanced IFN-I response in chimeric FOSL1 knockout (KO) mice after malaria parasite or vesicular stomatitis virus (VSV) infection.

**FIG 1:**
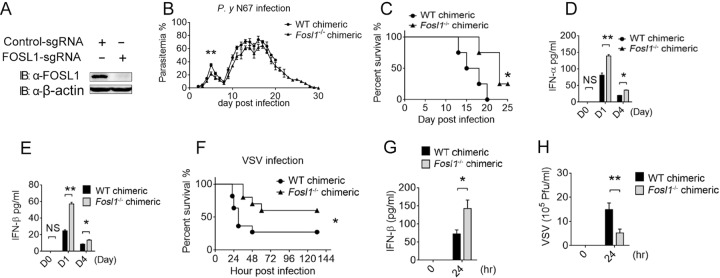
FOSL1 deficiency increases IFN-I levels and resistance to malaria or vesicular stomatitis virus (VSV) infection. (A) Immunoblot analysis of FOSL1 knockout efficiency in bone marrow cells of *Fosl1*^*−/−*^ chimeric mice. IB, immunoblot. (B) Daily parasitemia of wild-type (WT) and *Fosl1*^*−/−*^ chimeric mice infected with *Plasmodium yoelii nigeriensis* N67 (*P. y* N67). (C) Survival rates of WT mice and *Fosl1*^*−/−*^ chimeric mice infected with the N67 parasite. (D and E) IFN-α (D) and IFN-β (E) levels in sera of WT and *Fosl1*^*−/−*^ chimeric mice at day 1 (D1) and D4 postinfection with the N67 parasite. (F) Survival rates of WT and *Fosl1*^*−/−*^ chimeric mice intravenously injected with VSV (5 × 10^8^ PFU/g). (G) Enzyme-linked immunosorbent assay (ELISA) accessing the level of IFN-β production in sera of WT and *Fosl1*^*−/−*^ chimeric mice at 0 h and 24 h after VSV infection. (H) The viral titer in the blood at 0 h and 24 h after infection with VSV, assessed using plaque assay (38). The genetic background of the WT and *Fosl1*^*−/−*^ chimeric mice (from The Jackson Laboratory) is C57BL/6J, and the protocol of generation of chimeric mice is described in Materials and Methods. At least five mice were used in each group. *, *P* < 0.05; **, *P* < 0.01; ***, *P* < 0.001; NS, not significantly different from control group (Student’s *t* test). Data are representative of results of three independent experiments (means ± SD in panels B to H).

From a genome-wide transspecies expression quantitative trait locus (ts-eQTL) screen, we previously identified a large number of putative regulators of IFN-I signaling, including FOSL1, which appears to negatively regulate IFN-I in response to malaria parasite infection (21). To investigate the functional importance of FOSL1 in regulating innate immune responses in malaria, we first generated chimeric FOSL1 KO mice by reconstituting irradiated recipient mice with *Fosl1* KO bone marrow cells using CRISPR/Cas9. The *Fosl1* gene KO efficiency in the chimeric mice was verified using Western blot analysis (Fig. 1A). After infection with *Plasmodium* N67 parasites, we found that the Fosl1^−/−^ chimeric mice had lower parasitemia levels and longer host survival times than the control wild-type (WT) mice (Fig. 1B and C). Significantly larger amounts of IFN-α and IFN-β were also observed in the sera of Fosl1^−/−^ chimeric mice than in the sera of control mice, particularly at day 1 (h 24) postinfection (Fig. 1D and E). The levels of IFN-α and IFN-β mRNA in the spleen of the Fosl1^−/−^ chimeric mice were also significantly increased after parasite infection (see Fig. S1A and B in the supplemental material). With the increased expression of IFN-I in the spleens of mice infected with N67 parasite or VSV, *fosl1* transcript levels were also increased at day 1 and day 4 postinfection (Fig. S1C and D). The high IFN-I level at day 1 postinfection might contribute to the lower parasitemia at day 5 and the better survival rate for the Fosl1^−/−^ chimeric mice. These results suggest that FOSL1 plays an important negative role in IFN-I production during malaria.

10.1128/mBio.02161-16.1 Figure S1  FOSL1 deficiency promotes the activation of IFN-I *in vivo*. (A and B) Real-time qPCR analysis of IFN-α and IFN-β mRNA levels in the spleen of WT and FOSL1 knockout chimeric mice at day 1 and day 4 post N67 infection. (C and D) Fold changes in mRNA levels of IFN-β and *fosl1* in the splenic cells of mice infected with the N67 parasite (C) or with VSV (D). D0, day 0; D1, day 1; D4, day 4. *, *P* < 0.05; **, *P* < 0.01; ***, *P* < 0.001; NS, not significantly different from control group (Student’s *t* test). Data are representative of results of three independent experiments (means ± SD). Download Figure S1, TIF file, 0.2 MB. Copyright © 2017 Cai et al. 2017 Cai et al. This content is distributed under the terms of the Creative Commons Attribution 4.0 International license.

As a negative regulator of the IFN-I response, FOSL1 may also affect the host response to viral infections. To test this possibility, we challenged Fosl1^−/−^ chimeric and WT control mice with VSV and found that Fosl1^−/−^ chimeric mice were more resistant to VSV infection than the WT control mice (Fig. 1F). The Fosl1^−/−^ chimeric mice produced significantly higher levels of IFN-I in sera and had lower VSV titers in the blood than control chimeric mice 24 h after VSV infection (Fig. 1G and H). Taken together, these results suggest that FOSL1 deficiency enhances the production of IFN-I and antiviral immunity.

### Ectopic expression of FOSL1 suppresses IFN-I response.

**FIG 2:**
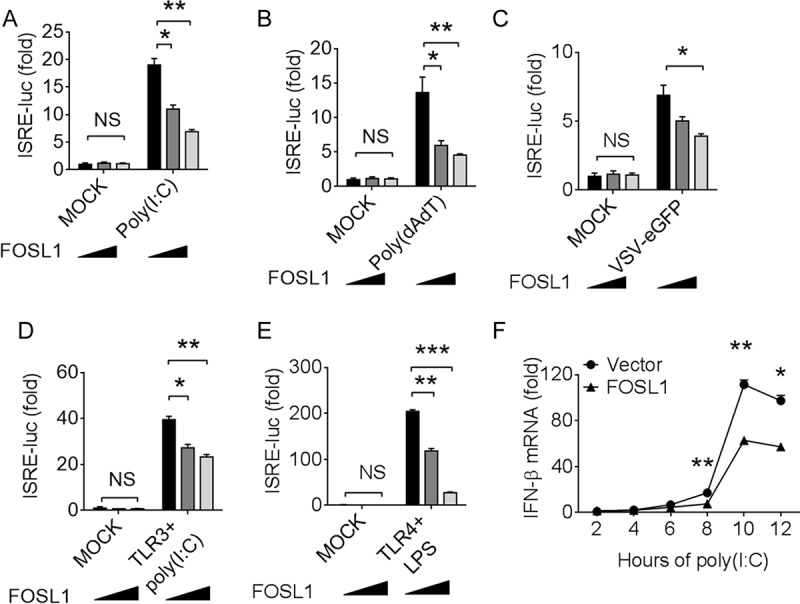
Ectopic expression of FOSL1 inhibits IFN-I signaling. (A to C) Luciferase signals in 293T cells after transfection with ISRE-luc reporter plasmid together with increasing amounts of FOSL1 overexpression plasmid (0, 150, and 300 ng), followed by treatment with poly(I:C) (1 µg/ml) (A), poly(dAdT) (1 µg/ml) (B), and GFP-tagged VSV (VSV-eGFP) (multiplicity of infection [MOI], 0.01) (C), respectively. (D and E) Luciferase signals in 293T/TLR3 and 293T/TLR4 cells after transfection with ISRE-luc reporter plasmid together with increasing amounts of FOSL1 overexpression plasmid, followed by treatment with poly(I:C) (1 µg/ml) (D) or LPS (1 µg/ml) (E). (F) Real-time qPCR analysis of IFN-β mRNA levels at different time points after poly(I:C) stimulation with or without overexpression of FOSL1. *, *P* < 0.05; **, *P* < 0.01; ***, *P* < 0.001; NS, not significantly different from control group (Student’s *t* test). Data are representative of results of three independent experiments (means ± SD in panels A to F).

To further demonstrate the role of FOSL1 in regulating the IFN-I response, we transfected 293T cells with increasing amounts (0, 150, and 300 ng) of FOSL1 expression vector, as well as plasmids containing a luciferase reporter driven by an ISRE (interferon-sensitive response element) promoter and renilla luciferase vector as internal control, and then stimulated the transfected cells with poly(I:C), poly(dAdT), or a green fluorescent protein (GFP)-tagged VSV (VSV-enhanced GFP [VSV-eGFP]). We found FOSL1 dosage-dependent inhibition of luciferase signals in all three treatments (Fig. 2A to C). Similar results were obtained from TLR3-293T cells (293T cells expressing TLR3) and TLR4-293T cells (293T cells expressing TLR4) after treatments with poly(I:C) and LPS, respectively (Fig. 2D and E). We also measured IFN-β mRNA levels in 293T cells with or without transfection of plasmid carrying the *Fosl1* gene and found significant reduction in IFN-β mRNA levels in FOSL1-overexpressing cells at 8 to 12 h after poly(I:C) stimulation (Fig. 2F). These results suggest that FOSL1 is a negative regulator of IFN-I signaling.

### Knockdown of FOSL1 enhances IFN-I production and inhibits viral replication.

**FIG 3:**
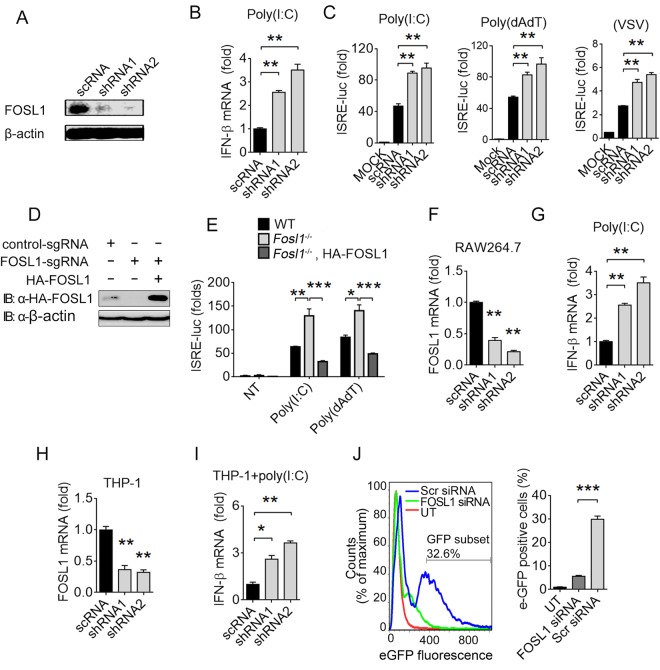
Knockdown of FOSL1 enhances IFN-I production and inhibits viral replication. (A) Reduced FOSL1 expression after shRNA knockdown as detected by Western blotting. (B) IFN-β mRNA in WT and FOSL1 knockdown 293T cells stimulated with poly(I:C) overnight. (C) Luciferase activities in 293T cells transfected with FOSL1-specific shRNAs or control shRNA (scRNA) together with ISRE luciferase reporter and then stimulated with poly(I:C), poly(dAdT), or GFP-tagged VSV (VSV-eGFP). (D) Western blot analysis of FOSL1 protein expression in WT, FOSL1 KO, and 293T cells overexpressing FOSL1. (E) ISRE luciferase signals in WT, FOSL1 KO, or FOSL1 KO 293T cells reconstituted with a plasmid expressing HA-tagged FOSL1 after poly(I:C) (1 µg/ml) or poly(dAdT) (1 µg/ml) stimulation overnight. ISRE-luc reporter plasmid was also cotransfected. NT, no treatment. (F and G) Real-time qPCR analysis of *Fosl1* mRNA after shRNA knockdown in RAW264.7 cells (F) and of IFN-β mRNA levels after treatment with poly(I:C) (G). (H and I) The same experiments as described for panels F and G but performed in THP-1 cells. (J) Flow cytometry assessing the infection of THP-1 cells treated with FOSL1-specific siRNA (FOSL1 siRNA) or scrambled siRNA (Scr siRNA) and then infected with VSV-eGFP with an MOI of 10. UT, uninfected treatment. *, *P* < 0.05; **, *P* < 0.01; ***, *P* < 0.001; NS, not significantly different from control group (Student’s *t* test). Data are representative of results of three independent experiments (means ± SD in panels B, C, and E to J).

To further investigate the role of FOSL1 in suppressing IFN-I response, we used short hairpin RNA (shRNA) to knock down endogenous FOSL1 in different cell types and then stimulated them with poly(I:C), poly(dAdT), or VSV-eGFP and measured IFN-β mRNA levels and/or luciferase activities driven by ISRE promoter. Compared with the control scramble shRNA (scRNA), FOSL1-specific shRNAs greatly reduced FOSL1 protein expression (Fig. 3A), which led to significantly increased IFN-β mRNA levels in 293T cells after stimulation with poly(I:C) (Fig. 3B). Significantly increased levels of ISRE-driven luciferase signals were also observed after poly(I:C) or poly(dAdT) treatment or VSV-eGFP infection (Fig. 3C). Meanwhile, ectopic expression of FOSL1 could restore the inhibition of ISRE-luc reporter activity induced by poly(I:C) or poly(dAdT) treatment (Fig. 3D and E), validating the inhibitory activity of FOSL1 in IFN-I signaling. Similarly, we observed increased IFN-β mRNA levels in FOSL1 knockdown RAW264.7 cells compared with control cells (Fig. 3F and G). The reduction in *Fosl1* mRNA levels also led to significant increase in IFN-β mRNA levels (Fig. 3H and I) and reduction of levels of VSV-eGFP-infected THP-1 cells (Fig. 3J). We also disrupted the *Fosl1* gene in splenocytes and showed that the IFN-β mRNA levels were significantly higher than those seen with the control group after VSV and iRBC stimulations (Fig. S2A and B). Similar results were observed in trophoblast cells after stimulations with VSV, iRBCs, poly(I:C), and parasite RNA (Fig. S2C). Taken together, these data demonstrate that FOSL1-specific knockdown can enhance IFN-I response and antiviral immunity.

10.1128/mBio.02161-16.2 Figure S2  Disruption of *Fosl1* gene expression in splenocyte and trophoblasts increased IFN-β transcript levels after stimulations. (A and B) Real-time qPCR analysis of IFN-β mRNA levels in wild-type (WT) and FOSL1 knockout (KO) splenocytes after stimulation with vesicular stomatitis virus (VSV) or infected red blood cells (iRBCs) at the indicated time points. (C) Real-time qPCR analysis of IFN-β mRNA levels in WT and FOSL1 KO trophoblast cells stimulated with VSV, poly(I:C), parasite RNA (pRNA), or infected RBCs (iRBCs) for 16 h. NT, no-treatment control. The *Fosl1* gene was disrupted using the LentiCRISPR/Cas9 system in splenocytes and trophoblast cells described in Materials and Methods. *, *P* < 0.05; **, *P* < 0.01; ***, *P* < 0.001; NS, not significantly different from control group (Student’s *t* test). Data are representative of results of three independent experiments (means ± SD in panels A to C). Download Figure S2, TIF file, 0.3 MB. Copyright © 2017 Cai et al. 2017 Cai et al. This content is distributed under the terms of the Creative Commons Attribution 4.0 International license.

### FOSL1 acts on molecules upstream of TBK1 and IRF3 in the IFN-I signaling pathway.

**FIG 4:**
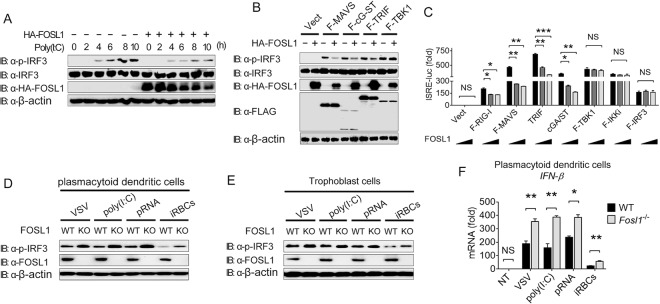
FOSL1 negatively regulates IFN-I signaling in different cell types. (A) Western blot analysis of phosphorylated IRF3 in 293T cells with or without overexpression of FOSL1 at different time points after poly(I:C) stimulation. (B) Western blot analysis of phosphorylated IRF3 in whole-cell lysates (WCL) of 293T cells after transfection with plasmids containing indicated genes encoding various adaptors, with or without overexpression of FOSL1. Vect, vector. (C) Luciferase activities in 293T cells transfected with ISRE-luc reporter plasmid together with plasmids expressing RIG-I, MAVS, cGAS plus STING, TRIF, TBK1, IKKi, or IRF3 and increasing amounts (0, 150, and 300 ng) of FOSL1 overexpression plasmid. (D) Western blot analysis of phosphorylated IRF3 in WT and FOSL1 KO plasmacytoid dendritic cells (pDCs) after stimulation with VSV, poly(I:C), parasite RNA (pRNA), or infected RBCs (iRBCs) for 16 h. (E) The same experiments as described for panel D but performed in trophoblast cells. (F) Real-time qPCR analysis of IFN-β mRNA levels in WT and FOSL1 KO pDCs stimulated with VSV, poly(I:C), parasite RNA (pRNA), or infected RBCs (iRBCs) for 16 h. No treatment (NT), control. The FOSL1 KO cells were generated using the LentiCRISPR/Cas9 system as described in Materials and Methods. *, *P* < 0.05; **, *P* < 0.01; ***, *P* < 0.001; NS, not significantly different from control group (Student’s *t* test). Data are representative of results of three independent experiments (means ± SD in panels C and F).

Because IRF3 is a transcription factor that plays a critical role in IFN-I responses to cytosolic RNA/DNA and virus infection (22, 23), we investigated the expression and phosphorylation of IRF3 with or without overexpression of FOSL1 and found that FOSL1 could inhibit phosphorylation of IRF3 but not the expression of IRF3 at the total protein level after poly(I:C) stimulation in 293T cells (Fig. 4A). FOSL1 overexpression reduced IRF3 phosphorylation induced by ectopic expression of MAVS or cGAS plus STING (cGAS/STING) or TRIF, but not that induced by TBK1 (Fig. 4B). Coexpression of RIG-I, MAVS, and cGAS plus STING with increasing amounts (0, 150, and 300 ng) of FOSL1 in 293T cells significantly reduced ISRE-luc signals, but the same result was not seen with TRIF, TBK1, IκB kinase (IKKi), or IRF3 (Fig. 4C). Similarly, increased phosphorylation of IRF3 in FOSL1 KO plasmacytoid dendritic cells (pDCs) or trophoblast cells was observed after stimulation with VSV, poly(I:C), parasite RNA (pRNA), or infected RBCs (iRBCs) (Fig. 4D and E), leading to increased IFN-β mRNA transcript levels in pDCs (Fig. 4F) and in trophoblasts (Fig. S2C). These results suggest that FOSL1 acts on molecules downstream of STING/MAVS/TRIF but upstream of TBK1/IRF3/IKKi in IFN-I signaling.

To determine whether overexpression of FOSL1 could affect transcriptional levels of key molecules in IFN-I pathways, we transfected the *Fosl1* gene into 293T cells and found no changes in transcriptional levels of RIG-I, MAVS, TBK1, IRF3, IRF7, TRAF3, or TRIF with or without poly(I:C) stimulation (Fig. S3A). No change in the total protein level of these molecules was found after overexpression of FOSL1 either (Fig. S3B). These results suggest that overexpression of FOSL1 could inhibit IFN-I signaling by affecting protein interaction or protein modification of signaling molecules but not by changing mRNA or protein levels of these key genes in the IFN-I pathway.

10.1128/mBio.02161-16.3 Figure S3  FOSL1 inhibits IFN-I responses through modification of signaling molecule upstream of TBK1. (A) Real-time qPCR analysis of RIG-I, MAVS, TBK1, IRF3, IRF7, TRAF3, and TRIF mRNA levels with or without poly(I:C) stimulation in the presence or absence of FOSL1 overexpression in 293T cells. (B) Western blot analysis of HA-tagged FOSL1 and FLAG-tagged selected IFN-I activators in 293T cells transfected with plasmids encoding the indicated activators with overexpression of FOSL1 (0, 150, and 300 ng for each of the three lanes). *, *P* < 0.05; **, *P* < 0.01; ***, *P* < 0.001; NS, not significantly different from control group (Student’s *t* test). Data are representative of results of three independent experiments (means ± SD in panel A). Download Figure S3, TIF file, 0.2 MB. Copyright © 2017 Cai et al. 2017 Cai et al. This content is distributed under the terms of the Creative Commons Attribution 4.0 International license.

### FOSL1 inhibits IFN-I response by interacting with TRAF3 and TRIF.

**FIG 5:**
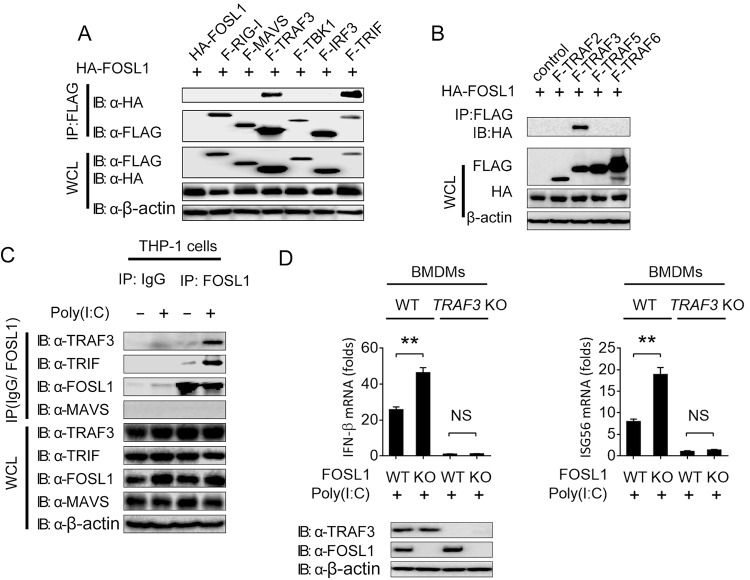
FOSL1 inhibits IFN-I response through modification of signaling molecules upstream of TBK1. (A) Immunoassay of 293T cell extracts transfected with plasmids encoding F-RIG-I, F-MAVS, F-TRAF3, F-TBK1, F-IRF3, and F-TRIF as well as HA-FOSL1, followed by immunoprecipitation (IP) with anti-FLAG beads and immunoblot analysis with anti-HA antibody. (B) Immunoassay of 293T cell extracts transfected with plasmids encoding F-TRAF2, F-TRAF3, F-TRAF5, and F-TRAF6 as well as HA-FOSL1, followed by IP with anti-FLAG beads and immunoblot analysis with anti-HA antibody. (C) IP and immunoblot analysis of THP-1 cell extracts treated with poly(I:C) or medium overnight. (D) Real-time qPCR analysis of IFN-β and ISG56 expression in WT, FOSL1 KO, TRAF3 KO, and FOSL1/TRAF3 double-knockout BMDMs after stimulation with poly(I:C) for 16 h. *, *P* < 0.05; **, *P* < 0.01; ***, *P* < 0.001; NS, not significantly different from control group (Student’s *t* test). Data are representative of results of three independent experiments (means ± SD in panel D).

To identify the key target molecules of FOSL1, we performed coimmunoprecipitation experiments and found that FOSL1 physically interacted with TRIF and TRAF3 but not with RIG-I, MAVS, TBK1, or IRF3 (Fig. 5A). No interaction between FOSL1 and TRAF2, TRAF5, or TRAF6 was detected (Fig. 5B). Furthermore, we found weak or no endogenous interaction of FOSL1 with TRAF3 or TRIF in immune cells (THP-1) without stimulation, but such interactions were increased after poly(I:C) stimulation (Fig. 5C). TRAF3 is an important regulator of IFN-I-dependent immune responses. To investigate whether the regulatory role of FOSL1 in IFN-I signaling is affected by TRAF3, we stimulated WT, TRAF3 KO, FOSL1 KO, and TRAF3/FOSL1 DKO (double-knockout) bone marrow-derived macrophages (BMDMs) with poly(I:C) and examined the mRNA expression of IFN-β and ISG56. We found that the upregulation of IFN-β and ISG56 in FOSL1 KO BMDMs was abolished in TRAF3/FOSL1 DKO BMDMs (Fig. 5D), suggesting that TRAF3 is required for FOSL1 in regulating IFN-I signaling. These results indicate that FOSL1 inhibits the IFN-I signaling by targeting TRAF3.

### FOSL1 inhibits the TRIF/TRAF3/TBK1 complex as well as K63 ubiquitination of TRAF3 and TRIF.

**FIG 6:**
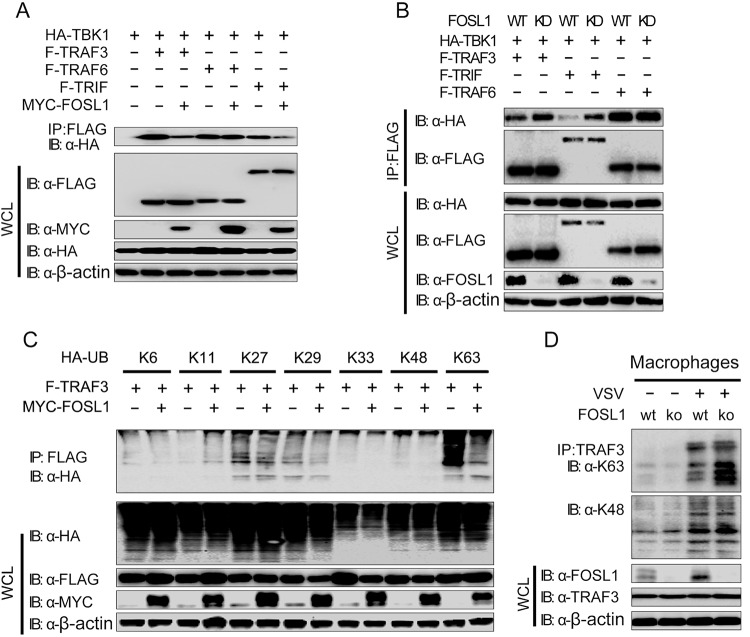
FOSL1 disrupts TBK1 and TRAF3/TRIF interactions and ubiquitination. (A) Cell lysates from 293T cells were transfected with expression plasmids for HA-TBK1, F-TRAF3, F-TRAF6, and F-TRIF with or without MYC-FOSL1, followed by immunoprecipitation (IP) with anti-FLAG and immunoblotting (IB) with antibodies against HA, FLAG, and MYC. (B) Cell lysates from 293T WT and FOSL1 knockdown cells were transfected with HA-TBK1, F-TRAF3, F-TRAF6, and F-TRIF, followed by IP with anti-FLAG and IB with antibodies against HA, FLAG, and endogenous FOSL1. (C) Cell lysates from 293T cells were transfected with expression plasmids for F-TRAF3 and different types of HA-ubiquitin (HA-UB) with or without overexpression of MYC-FOSL1. Co-IP for TRAF3 was performed using anti-FLAG breads and IB with antibodies against HA, FLAG, and MYC. (D) Cell lysates from WT and FOSL1 knockout macrophages (generated by the CRISPR/Cas9 technique) infected with VSV for 18 h (or left uninfected as a control) were subjected to coimmunoprecipitation using anti-TRAF3 antibody and detected with the indicated antibodies in Western blotting. Data are representative of three independent experiments with similar results.

The observations of physical associations of FOSL1 with TRIF and TRAF3 suggest that the inhibitory role of FOSL1 in IFN-I signaling could be mediated by interrupting the formation of TBK1/TRAF3 and/or TBK1/TRIF complexes. To further dissect the mechanism of how FOSL1 affects TRIF/TRAF3/TBK1 interaction and IFN-I signaling, we transfected various combinations of plasmids containing tagged TBK1, TRIF, TRAF3, or TRAF6 in 293T cells in the presence or absence of FOSL1 and found that interaction of TBK1 with TRAF3 or TRIF, but not with TRAF6, MAVS, IKKi, or IRF3, was reduced in the presence of ectopically expressed FOSL1 (Fig. 6A; see also Fig. S4A). Consistently, knockdown of FOSL1 could enhance the interaction of TBK1 with TRIF and TRAF3 but not that with TRAF6 (Fig. 6B). These results suggest that FOSL1 specifically disrupts the formation of the TBK1/TRAF3/TRIF complex.

10.1128/mBio.02161-16.4 Figure S4  Evaluation of FOSL1 interaction with molecules in type I interferon signaling. (A) FOSL1 did not affect TBK1 interactions with MAVS, IKKi, or IRF3 directly. Cell lysates from 293T cells were transfected with expression plasmids for HA-TBK1, F-MAVS, F-IKKi, and F-IRF3 with or without MYC-FOSL1, followed by immunoprecipitation (IP) with anti-FLAG and immunoblotting (IB) with antibodies against HA, MYC, and FLAG. (B) FOSL1 reduced TRIF K63 ubiquitination. 293T cells were transfected with expression plasmids for F-TRIF and different types of HA-UB with or without MYC-FOSL1. IP for TRIF was performed using anti-FLAG beads and IB with antibodies against HA, FLAG, and MYC. Data are representative of three independent experiments with similar results. Download Figure S4, TIF file, 0.3 MB. Copyright © 2017 Cai et al. 2017 Cai et al. This content is distributed under the terms of the Creative Commons Attribution 4.0 International license.

To determine how FOSL1 inhibits the interaction between TBK1 and TRAF3, we transfected 293T cells with hemagglutinin (HA)-tagged WT or mutant ubiquitins, along with FLAG-tagged TRAF3 with or without MYC-tagged FOSL1. Coimmunoprecipitation and immunoblot analysis showed that K63-linked polyubiquitination of TRAF3 was decreased in cells overexpressing FOSL1 (Fig. 6C), suggesting that FOSL1 affects K63-linked ubiquitination of TRAF3. Using a similar strategy, we also showed that FOSL1 inhibited K63 polyubiquitination of TRIF (Fig. S4B). To further confirm the role of FOSL1, we generated FOSL1 KO macrophages and showed increased K63 ubiquitination, but not K48-linked ubiquitination, of TRAF3 after VSV stimulation (Fig. 6D). These results suggest that FOSL1 interacts with TRAF3 and TRIF, leading to reduction of TRAF3 and TRIF K63 ubiquitination.

### FOSL1 expression and cytoplasmic translocation after stimulations.

**FIG 7:**
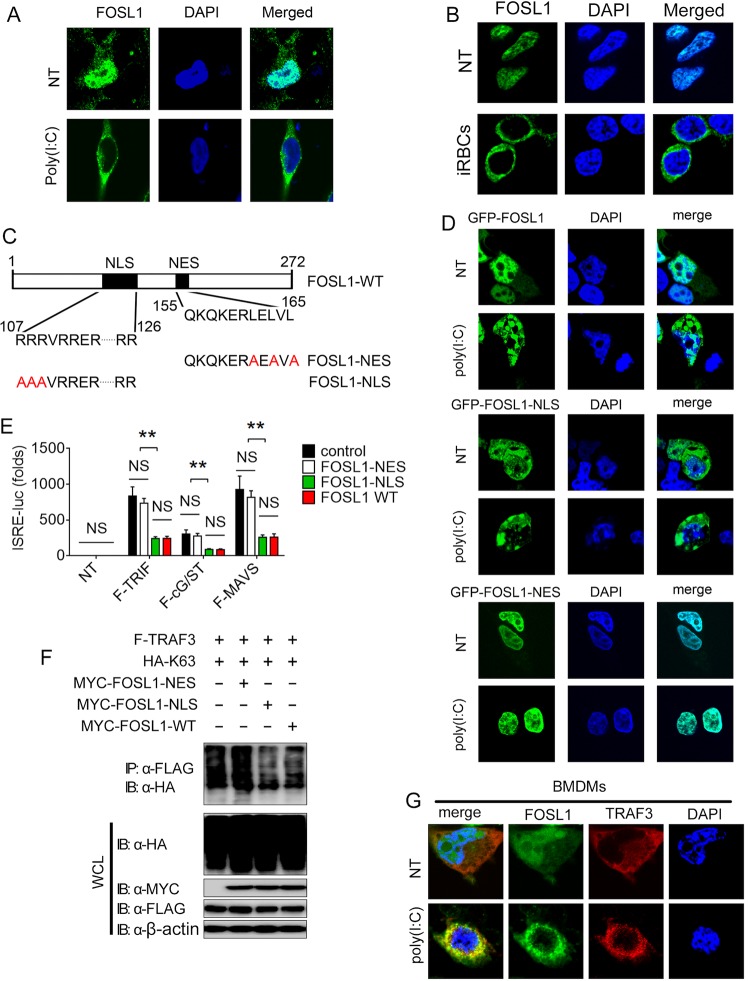
Virus infection induces FOSL1 expression and cytoplasmic translocation leading to inhibition of K63 ubiquitination of TRAF3. (A) Confocal microscopy analysis of FOSL1 in 293T cells with or without poly(I:C) stimulation; nuclei were detected with DAPI staining (blue). (B) Images of FOSL1 expression in bone marrow-derived macrophage (BMDM) after incubation with infected red blood cells (iRBCs) for 24 h. (C) A schematic diagram showing sequences of NLS/NES (nuclear localization signals/nuclear export signals) (predicted at http://www.psort.org/) of FOSL1 mutants. Amino acid substitutions in the NLS and NES motifs are indicated in red. (D) Confocal microscopy images of 293T cells transfected with plasmid containing GFP-tagged FOSL1-WT, GFP-tagged FOSL1-NES, or GFP-tagged FOSL1-NLS, followed by poly(I:C) stimulation or no stimulation (NT) overnight. (E) Luciferase activity in FOSL1 knockout 293T cells that were transfected with ISRE-luc reporter plasmid together with expression plasmids for MAVS, TRIF, or cGAS plus STING (cG/ST) as well as FOSL1 WT or mutants as indicated. (F) Cell lysates from FOSL1 KO 293T cells transfected with plasmids for FLAG-tagged TRAF3 and HA-tagged K63, as well as MYC-tagged FOSL1 or various MYC-tagged FOSL1 mutants, followed by IP with anti-FLAG and Western blot analysis with the antibodies as indicated. (G) Confocal microscopy analysis of FOSL1 and TRAF3 in BMDMs with or without poly(I:C) stimulation. Nuclei were detected with DAPI staining (blue). *, *P* < 0.05; **, *P* < 0.01; ***, *P* < 0.001; NS, not significantly different from control group (Student’s *t* test). Data are representative of results of three independent experiments (means ± SD in panel E).

Previous studies have shown that FOSL1 is dominantly localized in the nucleus (18), which raises the issue of how a nuclear protein known to function as a transcription factor could regulate interactions of TBK1 and TRIF/TRAF3 in IFN-I signaling in the cytoplasm. We hypothesized that FOSL1 may translocate from the nucleus to the cytoplasm after stimulation or viral infection, thus regulating the IFN-I pathway. To test this possibility, we used immunofluorescence microscopy to examine endogenous FOSL1 expression and localization. We found that FOSL1 was primarily expressed in the nucleus in unstimulated cells but was translocated to the cytoplasm after poly(I:C) treatment (Fig. 7A) or incubation with iRBCs (Fig. 7B). To investigate whether the cytoplasmic translocation of FOSL1 is required for its inhibition in IFN-I signaling pathway, we generated FOSL1 mutants with amino acid substitutions in the nuclear localization sequence (NLS) and nuclear export signal (NES) regions according to the prediction of PSORT (Fig. 7C). We found that FOSL1-NES protein lost the ability to translocate to the cytoplasm (i.e., remained in the nucleus). In contrast, FOSL1-NLS mutant protein remained in the cytoplasm with or without stimulation (Fig. 7D). We next generated FOSL1 KO 293T cells (Fig. S5A) using a CRISPR/Cas9 system and then introduced an expression vector containing FOSL1-WT, FOSL1-NES, or FOSL1-NLS, along with a plasmid containing TRIF, MAVS, or cGAS/STING. We found that both FOSL1-WT and FOSL1-NLS, but not FOSL1-NES, could inhibit the activity of an ISRE-luc reporter in FOSL1 KO 293T cells stimulated by TRIF, MAVS, or cGAS/STING overexpression (Fig. 7E). Additionally, both FOSL1-WT and FOSL1-NLS, but not FOSL1-NES, could reduce TRAF3 K63 ubiquitination (Fig. 7F).

10.1128/mBio.02161-16.5 Figure S5  Virus infection induces FOSL1 expression and cytoplasmic translocation. (A) Western blot analysis of FOSL1 protein expression in wild-type (WT) and FOSL1 KO 293T cells generated using the LentiCRISPR/Cas9 system. (B and C) Real-time qPCR analysis of FOSL1 mRNA in THP-1 cells (B, MOI = 1) and in peritoneal macrophages (pMs) (C) infected with vesicular stomatitis virus (VSV) at the indicated time points (MOI = 0.1, 1, and 10). (D) FOSL1 interacted with TRAF3 in the cytoplasm after VSV infection. Nuclear and cytoplasmic fractions from macrophages infected with VSV for 18 h or left uninfected as a control were subjected to immunoprecipitation (IP) with anti-TRAF3 antibody and Western blot analysis using the indicated antibodies. (E) FOSL1 knockout increased cytoplasmic TRAF3 K63 ubiquitination. Nuclear and cytoplasmic fractions from WT and FOSL1 KO BM-derived macrophages with or without VSV infection were subjected to IP using anti-TRAF3 antibody and detected using the indicated antibodies. The *Fosl1* gene was disrupted using the CRISPR/Cas9 KO system. **, *P* < 0.01; NS, not significantly different from control group (Student’s *t* test). Data are representative of results of three independent experiments (means ± SD in panels B and C). Download Figure S5, TIF file, 0.2 MB. Copyright © 2017 Cai et al. 2017 Cai et al. This content is distributed under the terms of the Creative Commons Attribution 4.0 International license.

The expression of FOSL1 was also increased in immune cells (THP-1 and peritoneal macrophages [pMs]) after infection with VSV (Fig. S5B and C). We next determined whether the cytoplasmic location of FOSL1 is required for its inhibitory function. Confocal microscope indicated that FOSL1 was translocated to cytoplasm and colocalized with TRAF3 in BMDMs after poly(I:C) stimulation (Fig. 7G). Similarly, we obtained the nuclear and cytoplasmic fractions, using methods previously described (24), and investigated endogenous protein interactions between FOSL1 and TRAF3 in the nuclear and cytoplasmic fractions in the cells with or without VSV stimulation. We found that FOSL1 was associated with TRAF3 in the cytoplasm, but not in the nuclear fraction, in macrophages after stimulation (Fig. S5D). Importantly, more K63-linked polyubiquitinated TRAF3 was observed in the cytoplasmic fractions of FOSL1 KO macrophages than in WT control cells, suggesting that FOSL1 deficiency enhances the K63-linked TRAF3 ubiquitination in the cytoplasm (Fig. S5E). Taken together, these results suggest that FOSL1 cytoplasmic localization is required for the inhibition of IFN-I signaling.

## DISCUSSION

Previous studies showed that FOSL1 is a member of the activator protein 1 (AP-1) family and is best known as a component of the AP-1 transcription factor complex (16). FOSL1 is a key downstream effector of the PI3K/AKT signaling pathway that operates by affecting the expression of genes associated with the invasive-vascular remodeling trophoblast phenotype and is responsible for the development of trophoblast lineages integral to establishing the mother-fetus interface (18). In addition, high expression of FOSL1 caused by rearrangement of the chromosome band at 11q12 appeared to be associated with desmoplastic fibroblastoma and directly induced MMP-1 and MMP-9 promoter activity in breast cancer progression (16, 20, 25). Recently, results of several studies have suggested that transcription factors or tumor regulator genes have a pivotal role in regulating innate immune signaling. For example, the SOX2 transcription factor has been identified as a bacterium-specific DNA sensor for the activation of TAK1 and TAB2 in neutrophils, which initiate antimicrobial innate immunity (26). The tumor suppressor protein PTEN controls the phosphorylation site of IRF3, resulting in enhanced nucleus translocation of IRF3 and antiviral innate immune signaling (27). The role of FOSL1 in the host IFN-I response has never been reported. In this report, we provide compelling evidence for the first time that FOSL1 inhibits IFN-I signaling by disrupting the interactions among TRAF3, TRIF, and TBK1.

By using a novel genome-wide transspecies expression quantitative trait locus (ts-eQTL) analysis, we found that FOSL1 was clustered with many genes that function as negative regulators in IFN-I pathways during malaria parasite infection (21), which suggest that it may play a role in the host IFN-I response. Our results show that FOSL1 functions as a new negative regulator in the IFN-I signaling pathway and the antiviral response. Ectopic expression of FOSL1 significantly reduced levels of VSV-induced ISRE promoter and transcription of IFN-β, whereas silencing of FOSL1 had opposite effects in different cell types. Hence, these results suggest that FOSL1 negatively regulates the production of IFN-I signaling. We further dissected the details of molecular interactions and mechanisms of FOSL1 in suppressing IFN-I response. Our data suggest that FOSL1 interacts with TRAF3 and TRIF in immune cells after viral infection. TRAF3, a major regulator of IFN-I production, serves as a critical link between TLR adaptors and downstream regulatory kinases (28). We found that FOSL1 interferes with the formation of TBK1 and the TRIF/TRAF3 complex and reduces K63 ubiquitination of TRAF3, leading to decreases in IRF3 phosphorylation and IFN-I production.

FOSL1 protein is known as a transcription factor and is normally expressed in the nucleus. Here we showed that FOSL1 was translocated into the cytoplasm after poly(I:C), VSV, or iRBC stimulations. Further studies showed that the FOSL1 NES mutant that lost its ability to translocate to the cytoplasm did not have the activity of inhibiting the IFN-I signaling pathway. Thus, the K63 ubiquitination of TRAF3 was impaired by FOSL1 in the cytoplasm. FOSL1 KO chimeric mice had significantly increased IFN-α and IFN-β production in the blood and spleen at day 1 and day 4 after malaria parasite infection, and IFN-I has been associated with inhibition of the growth of blood and liver stages of malaria parasites (13, 29, 30). Our results suggest that FOSL1 plays a negative role in IFN-I production and malaria protection. Indeed, FOSL1 KO chimeric mice had lower parasitemia or VSV titers and longer survival times after malaria parasite or virus infections. These *in vivo* studies provide further evidence that FOSL1 plays a critical role in the regulation of the innate immune response to malaria parasite and viral infection and reveal a previously uncharacterized function of FOSL1 in inhibiting IFN-I signaling.

On the basis of these results, we propose a working model to illustrate how FOSL1 negatively regulates the IFN-I response (see Fig. S6 in the supplemental material). Activation of TRIF-, cGAS-STING-, or RIG-I/MDA5-MAVS-mediated IFN-I pathways leads to production of IFN-I, which triggers a feedback mechanism with “translocation” of FOSL1 from the nucleus to the cytoplasm, where it inhibits the K63 ubiquitination of TRAF3 and TRIF and disrupts the formation of TBK1/TRAF3/TRIF complexes. The binding of FOSL1 to the TBK1 complexes, and the subsequent effects on ubiquitination of TBK1, results in reduced phosphorylation of IRF3 and suppression of IFN-I production. In contrast, blockade or disruption of FOSL1 expression or translocation increases IRF3 phosphorylation and IFN-I signaling.

10.1128/mBio.02161-16.6 Figure S6  Schematic showing putative mechanism of FOSL1 inhibiting type I interferon (IFN-I) pathways. Recognition of PAMPs or damage-associated molecular patterns (DAMPs) by TLRs, RIG-I, MDA5, or cGAS-STING activates IFN-I pathways that signal through the TRIF/TRAF3/TBK1 complex. Increased expression of FOSL1 in cytoplasm affects K63 ubiquitination of TRAF3 and TRIF and interferes with TBK1 binding to TRIF and TRAF3, leading to deceased phosphorylation of IRF3 and IFN-I signaling. Red arrows indicate decreased ubiquitination or phosphorylation. Blockade of FOSL1 translocation or disruption of FOSL1 expression leads to increased TARF3/TRIF ubiquitination, IRF3 phosphorylation, and IFN-I expression. Green arrows indicate increased ubiquitination or phosphorylation. Download Figure S6, TIF file, 0.5 MB. Copyright © 2017 Cai et al. 2017 Cai et al. This content is distributed under the terms of the Creative Commons Attribution 4.0 International license.

IFN-I plays a pivotal role in host immune responses to various pathogens, including viral, bacterial, and parasitic infections, but a chronic high level of IFN-I can also lead to pathological outcomes, including autoimmune diseases such as systemic lupus erythematosus (SLE) (31, 32). Chronic IFN-I signaling has been associated with hyperimmune activation and disease progression in persistent viral infections, while IFN-I blockade before and after establishment of persistent virus infection resulted in enhanced virus clearance (33, 34). Similarly, IFN-α/β enhances infection through inhibition of CD4^+^ T cell function during blood-stage infections by *Plasmodium berghei* and *Plasmodium chabaudi* (35). The appropriate level and timing of IFN-I production are critical for controlling infections without causing pathogenic effects. The discovery of FOSL1 inhibition of IFN-I responses by interfering with ubiquitination of TRIF and TRAF3 and the interaction of the two molecules with TBK1 could potentially be explored for developing strategies to modulate host IFN-I responses to infections. Several compounds have been reported to affect FOSL1 expression in different cells. For example, colon carcinoma cells treated with MEK inhibitor U0126 or PD184352 had lower FOSL1 expression (36). However, these compounds are not specific to FOSL1. New strategies are needed for identifying novel drugs to regulate FOSL1 expression in the future. Screening compound libraries for small molecules to block FOSL1 expression in cytoplasm or its interaction with TRAF3/TRIF/TBK1 may lead to therapies that can improve IFN-I response in malaria and other diseases. Interruption of these molecular interactions could be a potential therapeutic strategy for treatment of pathogen infection and, perhaps, autoimmune diseases.

## MATERIALS AND METHODS

### Ethics statement.

All mouse-related procedures were performed according to experimental protocols approved by the Animal Care and Welfare Committee at The Methodist Hospital Research Institute (Houston, TX) or according to protocol LMVR11E approved by the Animal Care and Use Committee in NIAID, National Institutes of Health.

### Cell lines, antibodies, and reagents.

Human 293T, THP-1, and mouse macrophage RAW 264.7 cell lines were cultured in Dulbecco’s modified Eagle’s medium (DMEM) or RPMI 1640 supplemented with 10% fetal bovine serum (FBS) and 1% antibiotic. Mouse BMDMs were generated by flushing bone marrow cells from the femurs and tibias of mice and were maintained in DMEM containing 10% FBS and 10% conditioned media from L929 cells overexpressing macrophage colony-stimulating factor (M-CSF). Poly(I:C), poly(dAdT), and LPS were from InvivoGen (San Diego, CA). Anti-MAVS (3393), anti-TRIF (4596S), anti-FOSL1 (5281), anti-TBK1 (3013), and anti-p-IRF3 (4947) were from Cell Signaling Technology, Inc. (Danvers, MA); Anti-K63 (05-1308) and anti-K48 (05-1307) were from EMD Millipore; anti-IRF3 (sc-9082), anti-TRAF3 (sc-6933), anti-FOSL1 (SC-605), and anti-GFP (sc-8334) were from Santa Cruz Biotechnology (Dallas, TX); anti-MYC-horseradish peroxidase (anti-MYC-HRP) (11814150001) was from Roche Applied Science (Indianapolis, IN); and anti-FLAG-HRP (M2) and anti-β-actin (A1978) were from Sigma (St. Louis, MO). Goat anti-rabbit IgG (H+L) Alexa Fluor 488 conjugate antibody (A-11034) was from Thermo Fisher Scientific. Protein G agarose used for immunoprecipitation was from Santa Cruz Biotechnology.

### Knockdown of FOSL1 using RNA interference.

FOSL1-specific and control (2-scramble mix) small interfering RNA (siRNA) oligonucleotides were purchased from Invitrogen and Integrated DNA Technologies, Inc. (Redwood City, CA). Two human and two mouse FOSL1-specific shRNA plasmids and control shRNA plasmids were obtained from Open Biosystems (Lafayette, CO). For plasmid transfection, 293T cells (1.5 × 10^5^) were plated in 24-well plates and transfected with plasmids using Lipofectamine 2000 (Invitrogen). siRNA was transfected into THP-1 cells using Nucleofector kit V (Invitrogen). Mouse shRNAs were introduced into BMDMs and RAW cells using lentiviral vectors.

### Luciferase activation assays.

Cells (1.5 × 10^5^) from different cell lines were transfected in a 24-well plate with ISRE firefly luciferase and pRL-TK renilla luciferase plasmids together with other plasmids containing genes of interest using Lipofectamine 2000. The cells were lysed and measured for luciferase activity using a dual-luciferase assay kit from Promega (Madison, WI) at the indicated time points after stimulation with various agents.

### Real-time qPCR analysis.

Total RNA was isolated from cells using TRIzol reagent (Invitrogen). First-stand cDNA was prepared from total RNA using a SuperScript III cDNA synthesis kit (Invitrogen). Real-time quantitative PCR (qPCR) was performed using SYBR green mix and procedures provided by the manufacturer (Invitrogen).

### Western blotting and immunoprecipitation.

Whole-cell lysates were separated on 10% SDS-PAGE, and proteins were transferred to nitrocellulose (NC) membranes for 1.5 h. The membranes were blocked with blocking buffer containing 5% nonfat milk–phosphate-buffered saline (PBS) at room temperature (RT) for 1 h before incubation with the primary antibodies at 4°C overnight with gentle shaking. After three washes performed with washing buffer (for 10 min each time), secondary HRP-conjugated antibodies were applied to the membranes at RT for 1 h. After the washes, the protein bands were visualized with chemiluminescent reagents following the manufacturer’s instructions (Millipore, Billerica, MA). For immunoprecipitation, FLAG- or HA-tagged protein agarose (Santa Cruz Biotechnology) was added into cell lysates (1:10 [vol/vol]) and incubated at 4°C on a shaker overnight. After four washes, the beads were eluted using protein loading buffer followed by boiling for 10 min before Western blot analysis.

### Molecular cloning and construction of plasmids.

The overexpression entry plasmid for FOSL1 (DQ891054) was obtained from Thermo Fisher Scientific (Waltham, MA) and was subsequently cloned into pcDNA-HA, pcDNA-FLAG, and pEGFP-C2 vectors. The plasmids containing the deletion domains of human FOSL1 were generated using PCR with the primers listed in Table S1 in the supplemental material.

10.1128/mBio.02161-16.7 Table S1  Primers used in this study. Download Table S1, DOCX file, 0.02 MB. Copyright © 2017 Cai et al. 2017 Cai et al. This content is distributed under the terms of the Creative Commons Attribution 4.0 International license.

### Immunofluorescence microscopy.

Cells were cultured on coverslips, fixed with 4% (wt/vol) paraformaldehyde–PBS for 15 min, and then permeabilized with 0.2% (vol/vol) Triton X-100. Primary antibodies (anti-FOSL1; 5281) were incubated at 4°C overnight after blocking with 3% bovine serum albumin (BSA)–PBS was performed. After three washes with PBS, the cells were then incubated with fluorochrome-conjugated secondary antibodies at RT for 1 h. Following three washes, the cells were stained, sealed with Vectashield antifade mounting medium with DAPI (4′,6-diamidino-2-phenylindole; Vector Laboratories, Burlingame, CA), and imaged using an Olympus 1X71S1F fluorescence microscope.

### Generation of FOSL1 KO mice using CRISPR-Cas9 genome editing.

The vector of a CRISPR-Cas9 FOSL1-sgRNA (FOSL1 single-guide RNA) construct and lentiviral particle was prepared according to methods described previously (37). CRISPR/Cas9 lentiviruses were generated by cotransfection of 293T cells with lentiviral vectors, pMD2.G, psPAX2, and CRISPR/Cas9-FOSL1-sgRNA or CRISPR/Cas9-V2 as a control. Bone marrow cells from femurs and tibias of 6-to-8-week-old female C57BL/6 mice were isolated, transduced with concentrated CRISPR/Cas9 lentiviral vector in the presence of 2 μg/ml Polybrene, and cultured overnight. The cells were harvested and intravenously injected into irradiated (950 cGy) 6-to-8-week-old female C57BL/6 mice. The mice were infected with malaria parasites or VSV 6 to 8 weeks after reconstitution. The efficiency of gene disruption was monitored using immunoblot analysis of FOSL1 expression in bone marrow cells of the chimeric mice.

### Statistical analysis.

Data are represented as means ± standard deviations (SD) where indicated, and Student’s *t* test was used with GraphPad Prism 5.0 software for all statistical analyses. Differences between groups were considered significant when the *P* value was <0.05.
