# Supplementary figures and images for: FOSL1 Inhibits Type I Interferon Responses to Malaria and Viral Infections by Blocking TBK1 and TRAF3/TRIF Interactions
Source: mBio. 2017 Jan 3;8(1):e02161-16. doi: 10.1128/mBio.02161-16 (PMC5210502; doi:10.1128/mBio.02161-16)

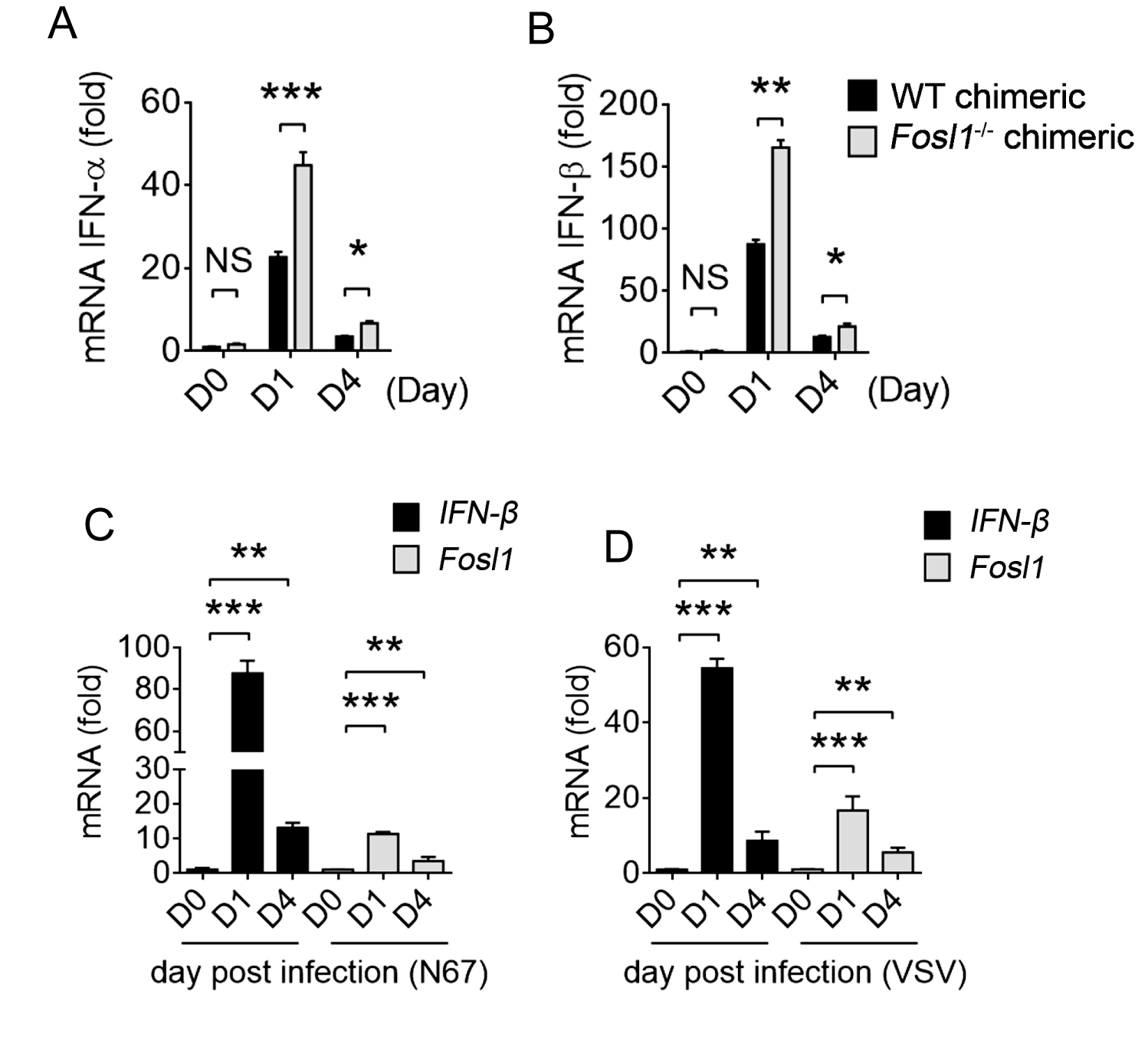

Supplement: Figure S1 [file mbo006163132sf2.tif]

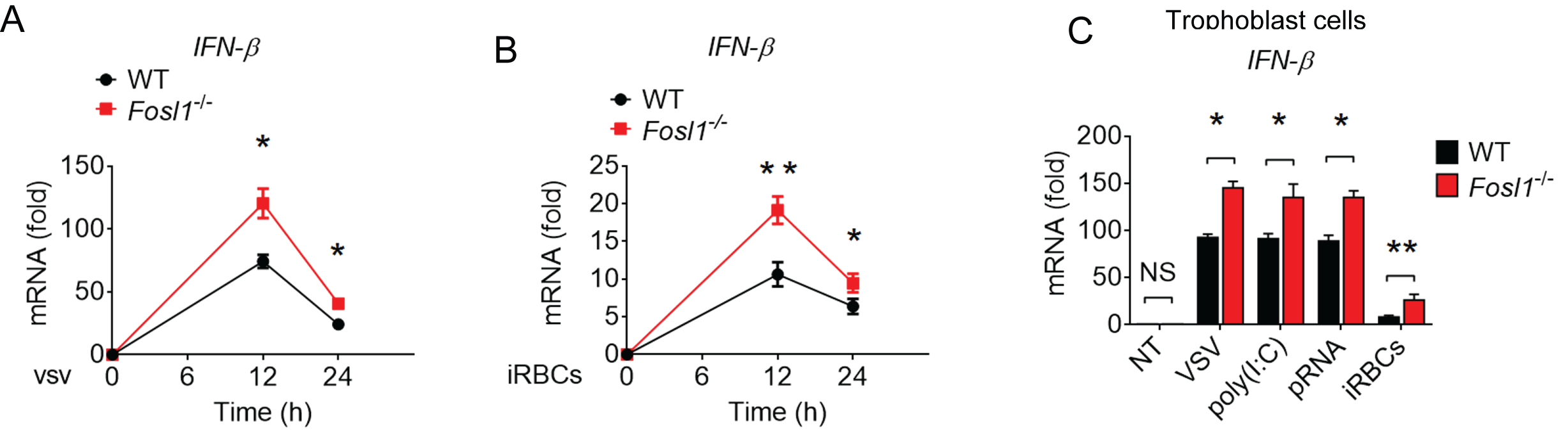

Supplement: Figure S2 [file mbo006163132sf3.tif]

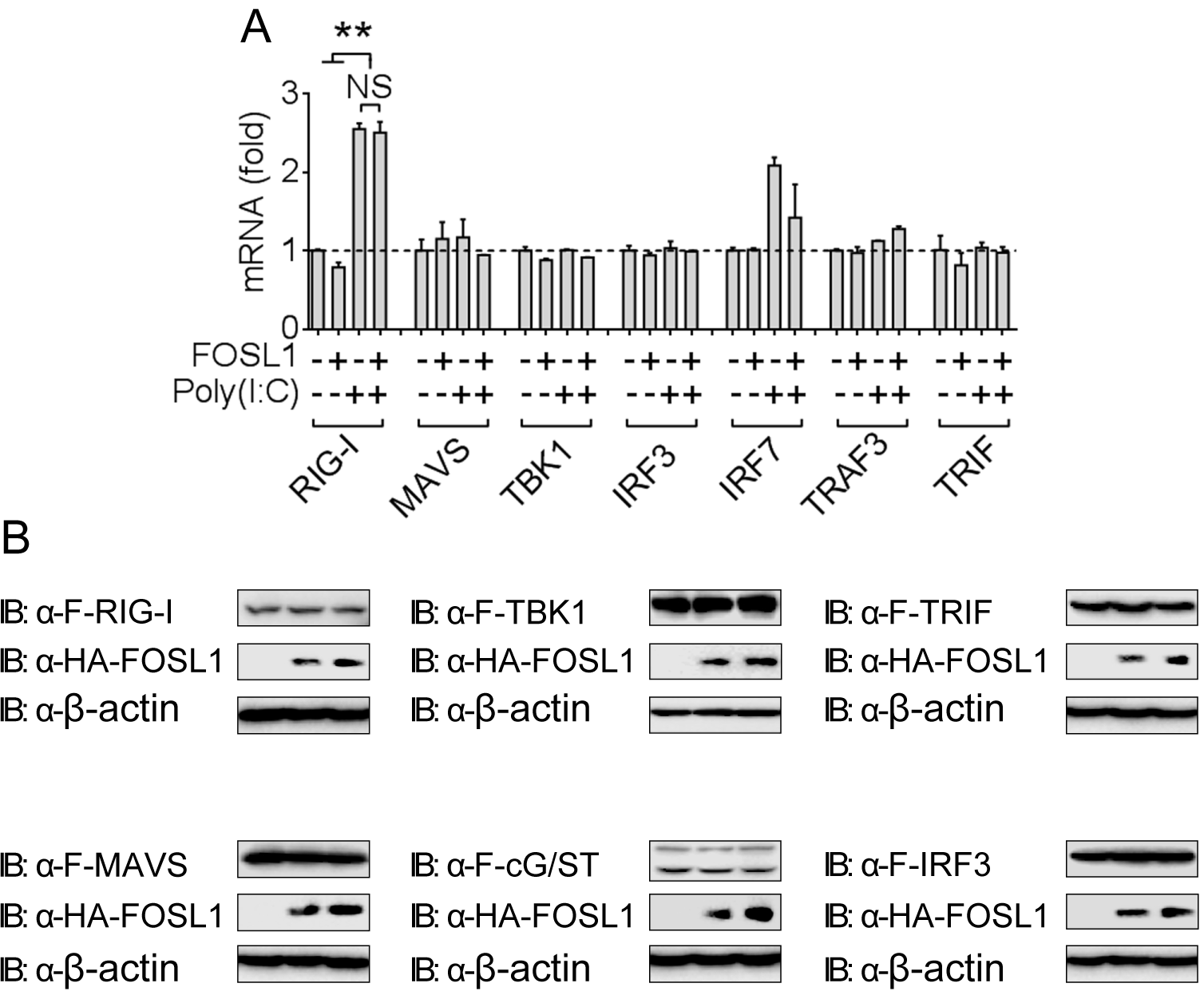

Supplement: Figure S3 [file mbo006163132sf4.tif]

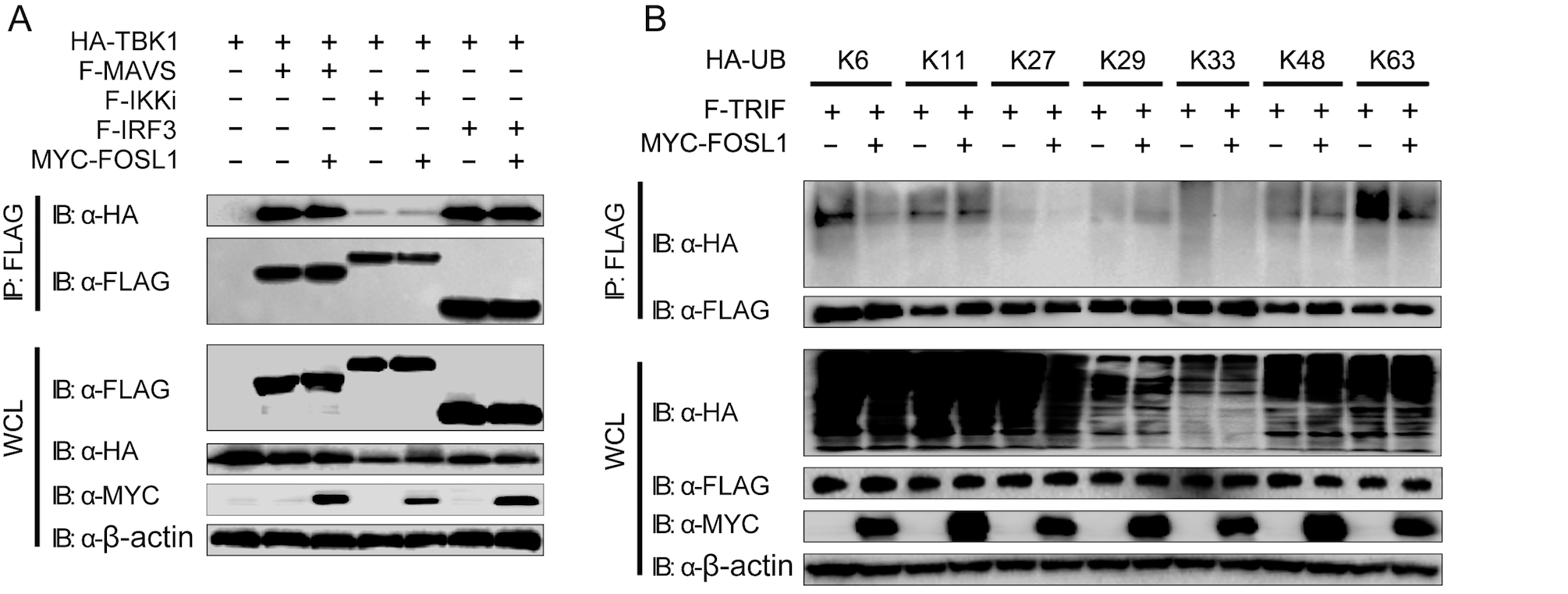

Supplement: Figure S4 [file mbo006163132sf5.tif]

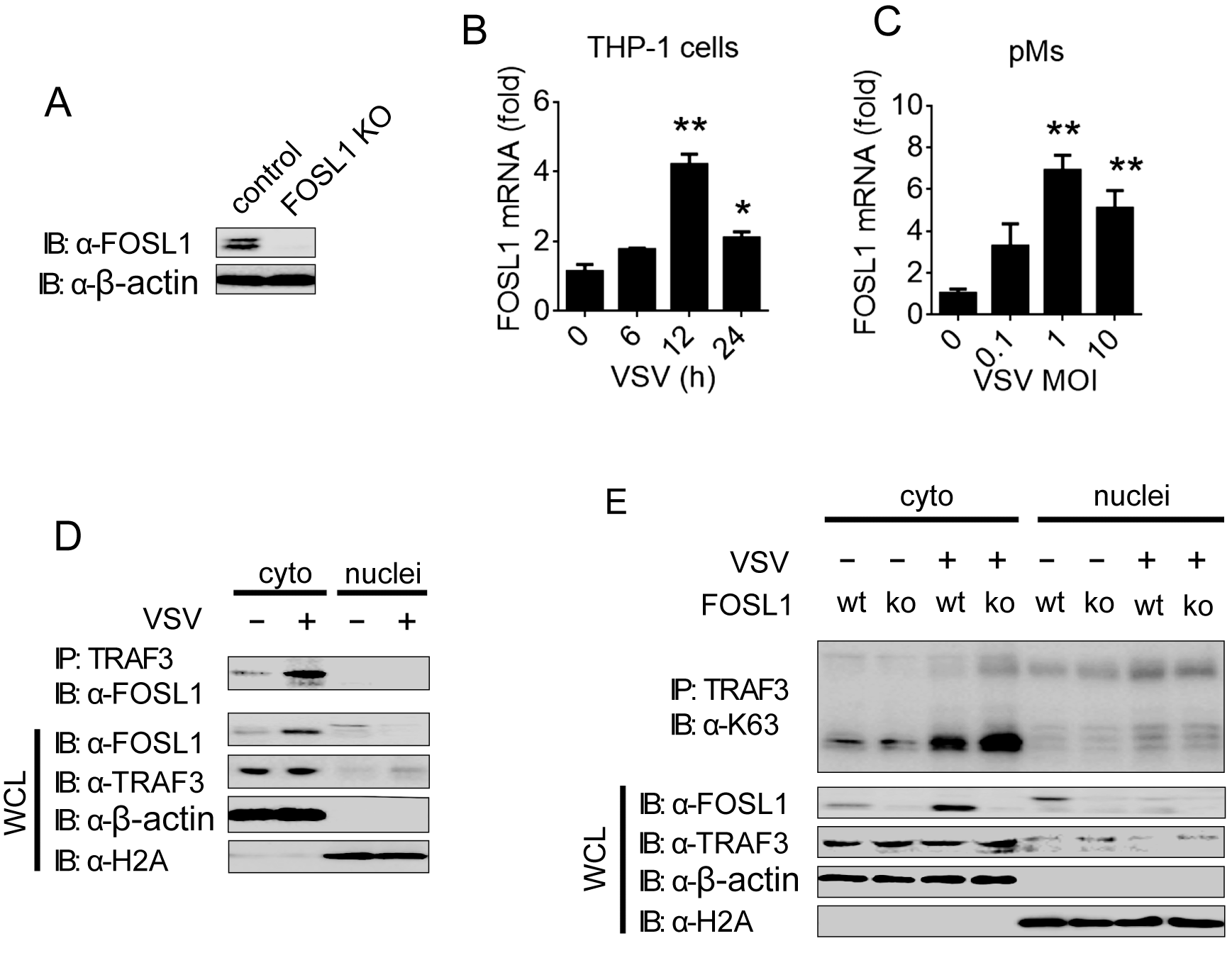

Supplement: Figure S5 [file mbo006163132sf6.tif]

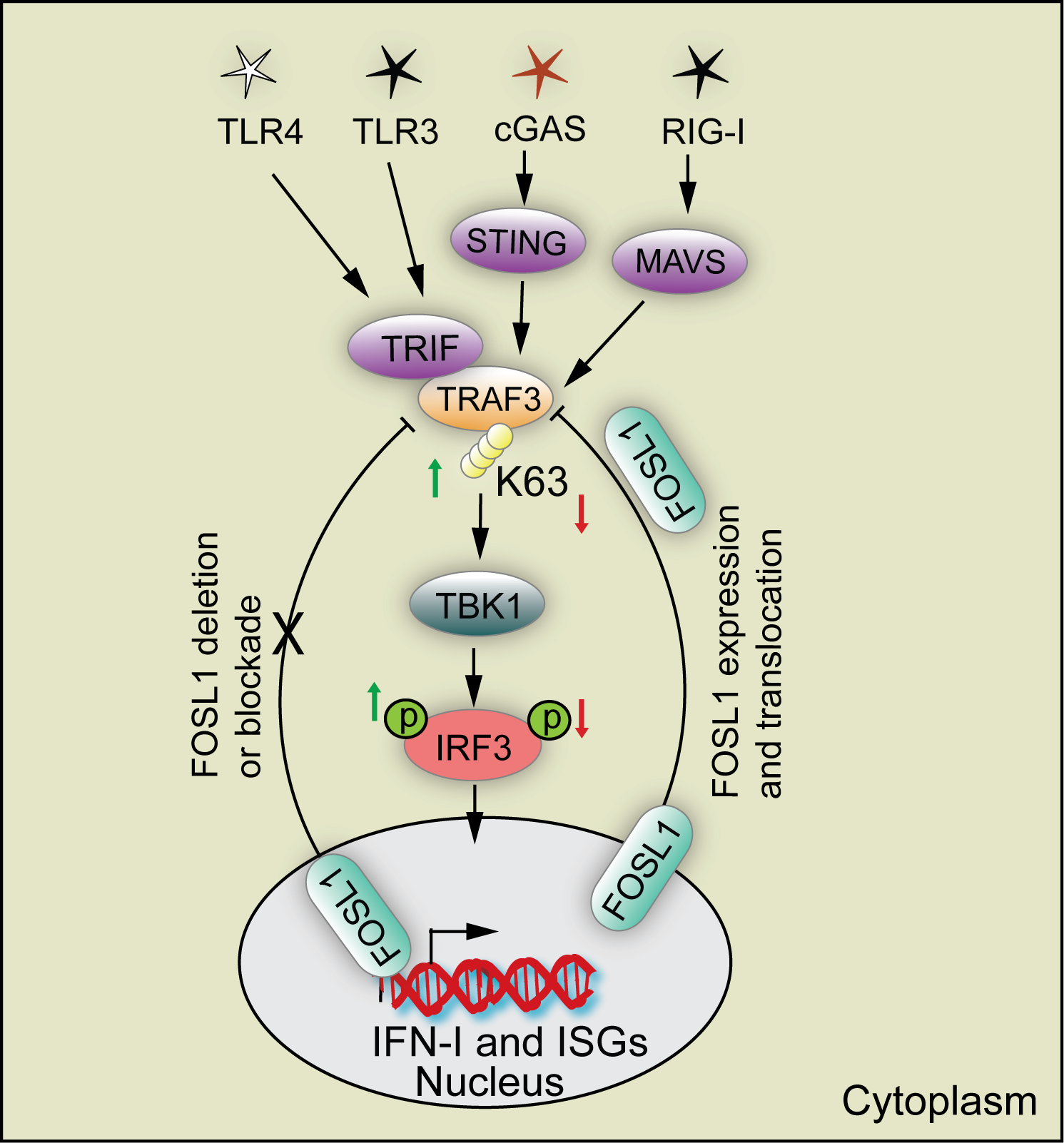

Supplement: Figure S6 [file mbo006163132sf7.tif]
