# Supplementary material for: FOSL1 Inhibits Type I Interferon Responses to Malaria and Viral Infections by Blocking TBK1 and TRAF3/TRIF Interactions
Source: mBio. 2017 Jan 3;8(1):e02161-16. doi: 10.1128/mBio.02161-16 (PMC5210502; doi:10.1128/mBio.02161-16)
Supplement: Table S1 [file mbo006163132s1.docx]

**Table S1. Primers used in this study.**

| Primers for domain | hFOSL1(DBD) forward primer,5'TTCCGAGACTTCGGGGA |
| --- | --- |
|  | hFOSL1(DBD) reverse primer,5'GTCGGTCAGTTCCTTCCT |
|  | hFOSL1(LZD) forward primer,5'TTCCTGCAGGCGGAGACT |
|  | hFOSL1(LZD) reverse primer,5'CAAAGCGAGGAGGGTTGG |
| mutants of FOSL1 | hFOSL1mF,GGGGACAAGTTTGTACAAAAAAGCAGGCTTCATGTTCCGAGACTTCGG |
|  | hFOSL1mR,GGGGACCACTTTGTACAAGAAAGCTGGGTCCTACTACAAAGCGAGGAGGGT |
|  | hFOSL1NLSmF,CAGCCCGGAGGAAGAGGAGGCTGCTGCAGTAAGGCGCGAGCGGAACAA |
|  | hFOSL1NLSmR,TTGTTCCGCTCGCGCCTTACTGCAGCAGCCTCCTCTTCCTCCGGGCTG |
|  | hFOSL1NESmF,AGAAGCAGAAGGAGCGCGCTGAGGCTGTGGCAGAAGCCCACCGACCCATC |
|  | hFOSL1NESmR,GATGGGTCGGTGGGCTTCTGCCACAGCCTCAGCGCGCTCCTTCTGCTTCT |
| primers for sgRNA | hFOSL1-sgRNA-F,CACCGCAACACCATGAGTGGCAGTC |
|  | hFOSL1-sgRNA-R,AAACGACTGCCACTCATGGTGTTGC |
|  | mFOSL1-sgRNA-F,CACCGGGCTGCGCGGGGCGACCGTA |
|  | mFOSL1-sgRNA-R,AAACTACGGTCGCCCCGCGCAGCCC |
| q-PCR | hIFN-β forward primer,5'CATTACCTGAAGGCCAAGGA |
|  | hIFN-β reverse primer,5'CAATTGTCCAGTCCCAGAGG |
|  | hGAPDH forward primer,5'TCAAGAAGGTGGTGAAGCAG |
|  | hGAPDH reverse primer,5'GAGGGGAGATTCAGTGTGGT |
|  | hFOSL1 forward primer,5'CGGAGACTGACAAACTGG |
|  | hFOSL1 reverse primer,5'CTCAGGTTCAAGCACAGG |
|  | mFOSL1 forward primer,5'ATGTACCGAGACTACGGGGAA |
|  | mFOSL1 reverse primer,5'CTGCTGCTGTCGATGCTTG |
|  | mIFN-β forward primer,5'TGCTCTCCTGTTGTGCTTCTCC |
|  | mIFN-β reverse primer,5'CATCTCATAGATGGTCAATGCGG |
|  | mGAPDH forward primer,5'TGGATTTGGACGCATTGGTC |
|  | mGAPDH reverse primer,5'TTTGCACTGGTACGTGTTGAT |
